# Association between mechanical ventilation parameters and mortality in children with respiratory failure on ECMO: a systematic review and meta-analysis

**DOI:** 10.3389/fped.2024.1302049

**Published:** 2024-01-16

**Authors:** Jaime Fernandez-Sarmiento, Maria Camila Perez, Juan David Bustos, Lorena Acevedo, Mauricio Sarta-Mantilla, Jennifer Guijarro, Carlos Santacruz, Daniel Felipe Pardo, Daniel Castro, Yinna Villa Rosero, Hernando Mulett

**Affiliations:** ^1^ Department of Critical Care Medicine and Pediatrics, Universidad de La Sabana, Fundación Cardioinfantil-Instituto de Cardiología, Bogotá, Colombia; ^2^ Department of Anesthesia and Cardiovascular Surgery, Fundación Cardioinfantil-Instituto de Cardiología, Bogotá, Colombia; ^3^ Department of Critical Care Medicine and Pediatrics, Universidad Nacional de Colombia, Bogotá, Colombia

**Keywords:** mortality, ECMO, children, respiratory failure, ARDS, mechanical ventilation, pneumonia

## Abstract

**Background:**

In refractory respiratory failure (RF), extracorporeal membrane oxygenation (ECMO) is a salvage therapy that seeks to reduce lung injury induced by mechanical ventilation. The parameters of optimal mechanical ventilation in children during ECMO are not known. Pulmonary ventilatory management during this therapy may impact mortality. The objective of this study was to evaluate the association between ventilatory parameters in children during ECMO therapy and in-hospital mortality.

**Methods:**

A systematic search of PubMed/MEDLINE, Embase, Cochrane, and Google Scholar from January 2013 until May 2022 (PROSPERO 450744), including studies in children with ECMO-supported RF assessing mechanical ventilation parameters, was conducted. Risk of bias was assessed using the Newcastle-Ottawa scale; heterogeneity, with absence <25% and high >75%, was assessed using I^2^. Sensitivity and subgroup analyses using the Mantel-Haenszel random-effects model were performed to explore the impact of methodological quality on effect size.

**Results:**

Six studies were included. The median age was 3.4 years (IQR: 3.2–4.2). Survival in the 28-day studies was 69%. Mechanical ventilation parameters associated with higher mortality were a very low tidal volume ventilation (<4 ml/kg; OR: 4.70; 95% CI: 2.91–7.59; *p* < 0.01; *I*^2^: 38%), high plateau pressure (mean Dif: −0.70 95% CI: −0.18, −0.22; *p* < 0.01), and high driving pressure (mean Dif: −0.96 95% CI: −1.83, −0.09: *p* = 0.03). The inspired fraction of oxygen (*p* = 0.09) and end-expiratory pressure (*p* = 0.69) were not associated with higher mortality. Patients who survived had less multiple organ failure (*p* < 0.01).

**Conclusion:**

The mechanical ventilation variables associated with higher mortality in children with ECMO-supported respiratory failure are high plateau pressures, high driving pressure and very low tidal volume ventilation. No association between mortality and other parameters of the mechanical ventilator, such as the inspired fraction of oxygen or end-expiratory pressure, was found.

**Systematic Review Registration:**

https://www.crd.york.ac.uk/prospero/display_record.php?ID=CRD42023450744, PROSPERO 2023 (CRD42023450744).

## Introduction

In refractory respiratory failure, extracorporeal membrane oxygenation (ECMO) support is an increasingly used salvage therapy (1, 2). According to the records of the Extracorporeal Life Support Organization (ELSO), in the last five years, more than 100,000 people have received ECMO support, including patients with COVID-19 (2–4). Of these, more than 13,000 have been children, with an overall survival rate of 59%, slightly higher in those receiving respiratory ECMO (69%). Children with refractory respiratory failure without ECMO have an associated mortality rate greater than 90% (5–8). These patients, during ECMO support, should simultaneously receive invasive mechanical ventilation (MV) (9). Mechanical ventilation-induced pulmonary injury (VILI) and patient-induced self-inflicted injury (P-SILI) are ventilatory support-related complications that are associated with increased morbidity and mortality in children with acute respiratory distress syndrome (PARDS) (10, 11).

Mechanical ventilation-induced pulmonary injury can be produced by global or local overdistension as well as by cyclic closure and opening of alveolar units in PARDS (12). Additionally, the heterogeneous distribution of pulmonary perfusion and the presence of patient-ventilator asynchronies may favor the appearance of P-SILI (13). Many of these consequences are related to inappropriate mechanical ventilation parameters for the patient's condition (including children with ECMO) that magnify the inflammatory response and increase endothelial permeability (14). The occurrence of VILI can be minimized by adopting lung protection strategies (15). However, optimal lung protection strategies during pediatric and neonatal ECMO are unknown. The most recent ECMO ventilatory support guidelines are based on expert opinion (6, 9, 15). There are no controlled clinical trials evaluating the efficacy and safety of ventilatory parameters in children with MV and ECMO on mortality and clinical outcomes.

The Second Pediatric Acute Lung Injury Consensus Conference (PALICC-2) recently suggested considering ECMO support in patients with a potentially reversible cause of PARDS who do not have adequate gas exchange with MV protective strategies (15). However, the optimal mechanical ventilation strategy in terms of ventilatory modes and parameters during ECMO has not yet been defined (16–18). Inspired fractions of oxygen (FI02) greater than 50% after ECMO day three have been associated with higher mortality (46% vs. 22%; *p* = 0.001) than in patients with lower FI02 (19). Whether other parameters in mechanical ventilation in ECMO, such as tidal volume, end-expiratory pressure (PEEP), plateau pressure, or driving pressure, may be associated with worse outcomes in children has not been clearly defined (7, 20). In this review, we systematically sought to evaluate and summarize the main studies in children that describe the most commonly used mechanical ventilation parameters during ECMO therapy and their association with in-hospital mortality, organ failure, and complications.

## Methods

### Search strategy and selection criteria

A systematic search was conducted in the main databases without language or time restrictions from January 2013 until May 1, 2023. PubMed, Medline, EMBASE, the Cochrane Library, and Google Scholar were electronically searched. A prospective registration was made in PROSPERO (ID 450744 available at http://www.crd.york.ac.uk/PROSPERO). The list of publications, including reviews, was searched manually. The reference list of relevant studies was manually checked for additional publications that were useful for analysis. Only human research was included, and a report was made according to the PRISMA and MOOSE guidelines.

### Inclusion and exclusion criteria

We included studies in our analysis that met the following criteria:

(1) studies involving children aged one month to 18 years old with refractory respiratory failure requiring ECMO support,
(2) studies describing mechanical ventilation support parameters during ECMO therapy in the first three days of admission to PICU,
(3) studies with any methodological design as well as gray literature (published in abstracts of major critical care conferences, OpenMD and OpenGrey) or peer-reviewed articles that reported complete information on sample size, type of support in VMI and associated outcomes,
(4) In the case of multiple studies with similar databases or overlapping populations, we selected the most recent for analysis.

Studies that met the following criteria were excluded: animal models, preclinical studies, neonatal stage research (including preterm and diaphragmatic hernia patients), letters to the editor, narrative reviews, and studies without clear descriptions of outcomes.

### Study selection and data processing

Five independent reviewers (MCP, JDB, JG, MSM, JFS) searched the articles, including titles and abstracts, and determined the eligibility of the investigations. The inclusion criteria were applied, and a full-text review with data extraction was performed. If there were doubts about eligibility, a second group of researchers (LA, HM, YV, CS, DP, and DC) made the decision on whether to include the study. All relevant data were extracted independently from the included studies (JFS, MCP, and JDB). Data on the parameters of mechanical ventilation, ventilation mode, inspired fraction of oxygen (Fi02), end-expiratory pressure (PEEP), plateau pressure (Pplat), and driving pressure (DP) were extracted.

### Methodological quality assessment

The quality of the studies was determined in several respects. External validity considered the target population, random error, and generalizability of results. Internal validity included evaluation of the design, description of the methodology, and form of evaluation and control of possible biases. We had planned to apply the Cochrane Risk of Bias Scale, version 2.0, but found no clinical trials to include in our analysis. Risk of bias in observational studies was assessed using the Newcastle-Ottawa scale (21). The criteria for evaluating the studies were sample selection, comparability, and outcomes.

### Outcomes

The principal aim of our study was to evaluate the impact of the parameters used in mechanical ventilation support (tidal volume, PEEP, Fi02, plateau pressure, DP) in children with ECMO on in-hospital mortality. Secondary outcomes were the total duration of ventilation and the presence of associated multiple organ failure (MODS).

### Data synthesis and statistical analysis

A descriptive analysis of the variables included in the study was initially performed. To compare quantitative variables, if the studies reported them as median or interquartile range, these were converted to means and standard deviations (SD) using the method proposed by Wan et al. (22). The risk of mortality was assessed with odds ratio (OR) with its respective 95% confidence interval (95% CI). The meta-analysis was conducted with a randomized effects model. Heterogeneity was assessed with visual inspection of forest plots, *Q*-test, and *I*^2^ statistics. A value less than 25% was considered without heterogeneity, mild from 25% to 50%, moderate from 50% to 75%, and high greater than 75%. Inverse-variance meta-analyses were performed by applying a logit transformation to the outcome, and the DerSimonian–Laird method (23) was used to estimate the heterogeneity of variance. The factors of sample size and methodological quality (high or poor) that could explain the heterogeneity were subjected to a subgroup and sensitivity analysis. Publication bias was assessed with the funnel plot, the Egger test for continuous variables, and the Peter's test for dichotomous variables. A *p* < 0.05 was considered statistically significant (except for the Egger test, for which *p* < 0.1 was considered significant).

The statistical analyses were done using Review Manager Version 5.4 (ReVman 5.4—Cochrane IMS, Cochrane Library, Oxford, United Kingdom) software.

## Results

**Figure 1:**
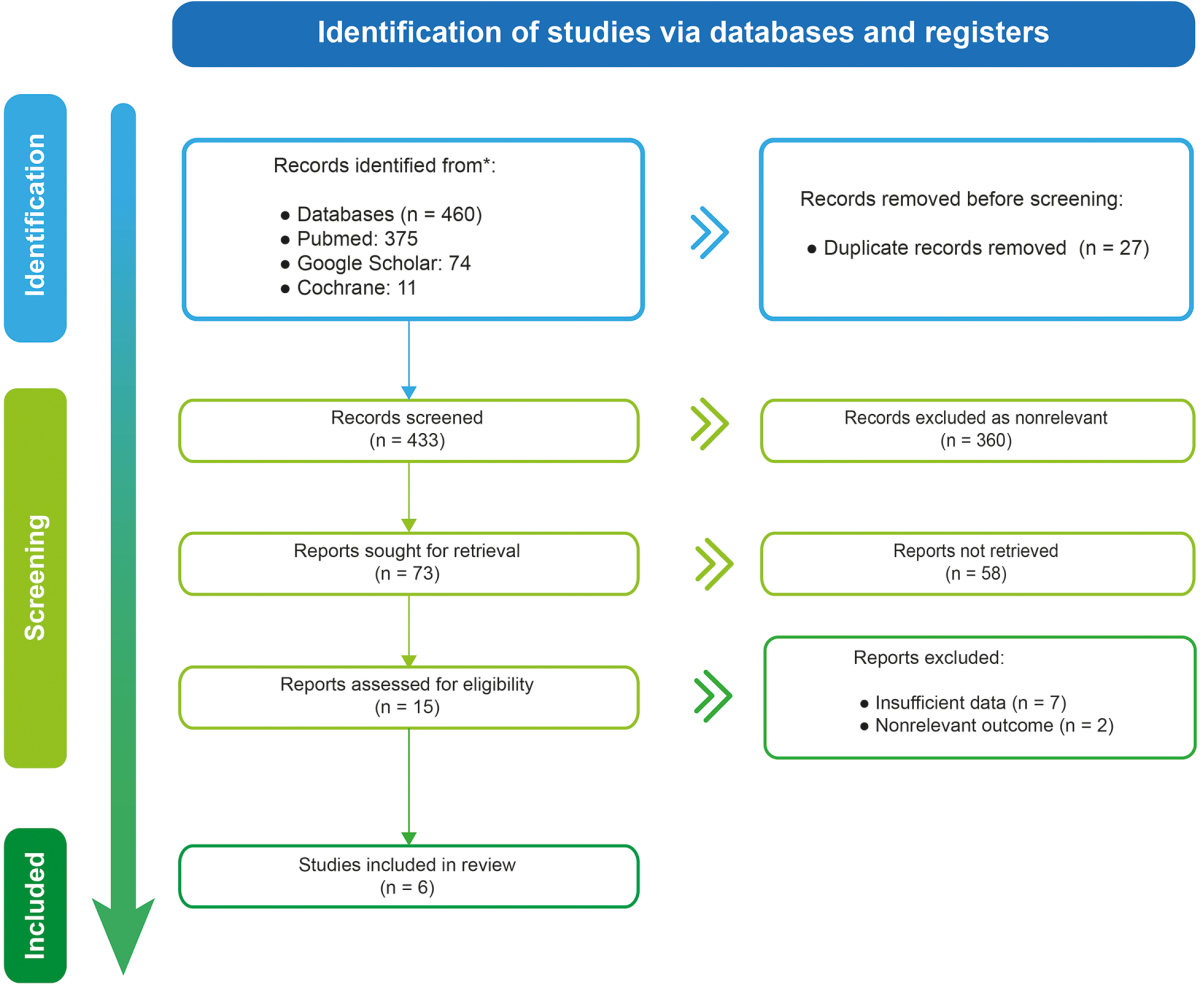
Aflowchart of study selection for meta-analysis.

A total of 460 studies were found (Figure 1). After removing duplicate or non-relevant studies, 15 studies were assessed for eligibility. A total of six studies with 1,512 patients met the inclusion criteria (7, 17, 19, 24–26). All the studies were observational. No clinical trials comparing the safety and efficacy of different mechanical ventilation support parameters with the outcomes of interest were found. Of the included studies, five were cohort, two were prospective (7, 17), and three were retrospective (20, 22, 24). One study was a case series of patients with leukemia (25). We obtained information on pressures and other ventilator settings in all studies. They are summarized in Table 1.

**Table 1:** General characteristics of the studies included in the meta-analysis.

Author	Country	Age (IQR) years	ECMO VV/VA (%)	N	Primary outcome	Mechanical ventilation mode used	Overall mortality	Primary cause of support	Sepsis %	% Patients with HFOV	Main cannulation site	ECMO duration (hours, IQR)
Barbaro et al. (17)	United States	4.2 (0.8,12)	100%	60	Hospital mortality at 90 days	No data	20%	Obstructive (25%) Pneumonia (46%) Sepsis (23%)	23%	56%	ND	No data
Friedman et al. (19)	United States	3.6 (1.1,12.1)	100%	180	Mortality	Conventional (66%–90.3% pressure targeted) Pressure release (12%) HFOV (4%) other HFV (4%) Extubated (1.5%)	26%	Respiratory syncytial virus (17%), other viruses (29%), bacterial pneumonia (13%), aspiration (6%), sepsis (4%), fungal pneumonia (2%), pertussis (1%)	4%	8%	ND	190 (117–337)
Friedman et al. (7)	United States	3.4 (0.9–12)	100%	1,161	Ventilatory support after ECMO start	Conventional (88%), HFOV (9%), other HFV (3%)	32%	No data	No data	9%	ND	197 (116–347)
Yun Cui et al. (24)	China	1.5 (0.8,3.2)	100%	18	In-hospital survival rate	Conventional (100%–100% pressure-targeted)	27.8%	Adenovirus pneumonia (100%)	No data	0%	Peripheral VV jugular—femoral	196 (152–309)
Zhang Yucai et al. (25)	China	4.4 (3.5,9.7)	100%	7	Clinical characteristics of leukemia patients with ARDS and ECMO requirement	Conventional protective (100%)	57.1%	ARDS due to bacterial, fungal, viral pneumonia 100%	No data	0%	Peripheral VA: 57%: Internal jugular—Right carotid. 29% Femoral. Peripheral VV	122 (56–166)
Miya et al. (26)	United States	1–18 years	51%/49%	86	In-hospital mortality	Conventional (76%–77% pressure-targeted), HFOV (7%), other (17%)	35%	Acquired heart disease (9.3%). Hypoxic lesion (2.3%) Immune dysfunction (12%). Congenital heart disease 8 (9.3%). Pertussis or sepsis (24%). Pneumonia or bronchiolitis (20%). Respiratory failure (81%). Neurological condition (3.5%)	28%	7%	ND	185 (108–309)

HFOV, high frequency oscillatory ventilation; HFV other, other high frequency ventilation modes; MV, mechanical ventilation; ECMO, extracorporeal membrane oxygentation; PEEP, Positive pressure at the end of expiration; Pp, Plateau presion; ND, not data.

**Figure 2:**
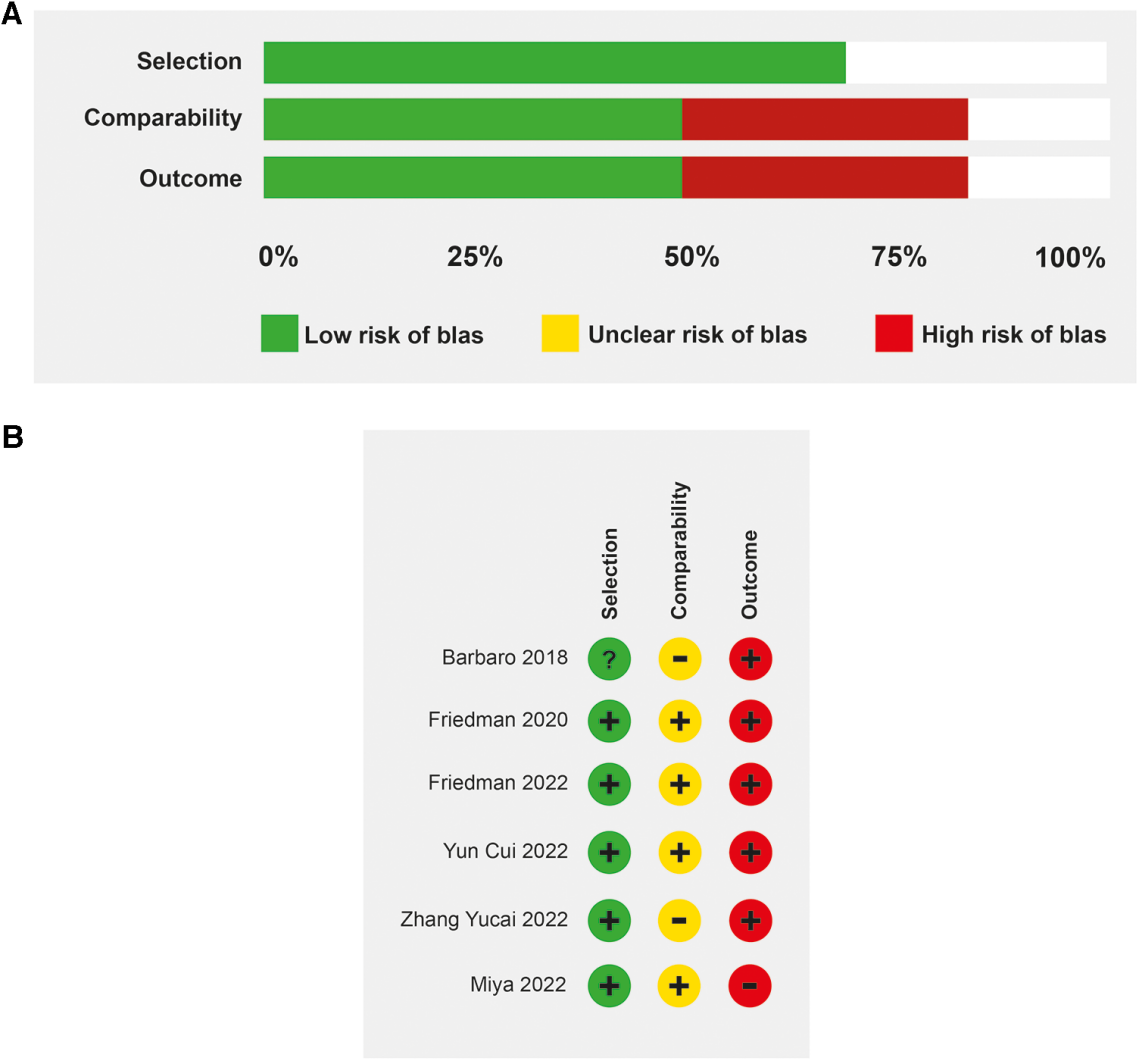
Risk of bias. (**A**) Evaluation of the risk of bias in the included studies on patients with mechanical ventilation and ECMO (**B**) Risk of bias determined by the Newcastle-Ottawa scale.

The median age in the included studies was 3.4 years (IQR: 3.2–4.2). In all studies, patients received veno-venous ECMO support, except in one study in which 57% of the patients had veno-arterial cannulation (25). The studies were at low to moderate risk of bias (Figures 2A,B). All prospective studies were classified as at moderate risk of bias. Some studies were single-arm and did not describe all ventilation parameters associated with mortality. The predominant source of information was medical record review and was not shown to be valid or reliable.

### Outcomes

#### Mortality associated with mechanical ventilation parameters

**Figure 3:**
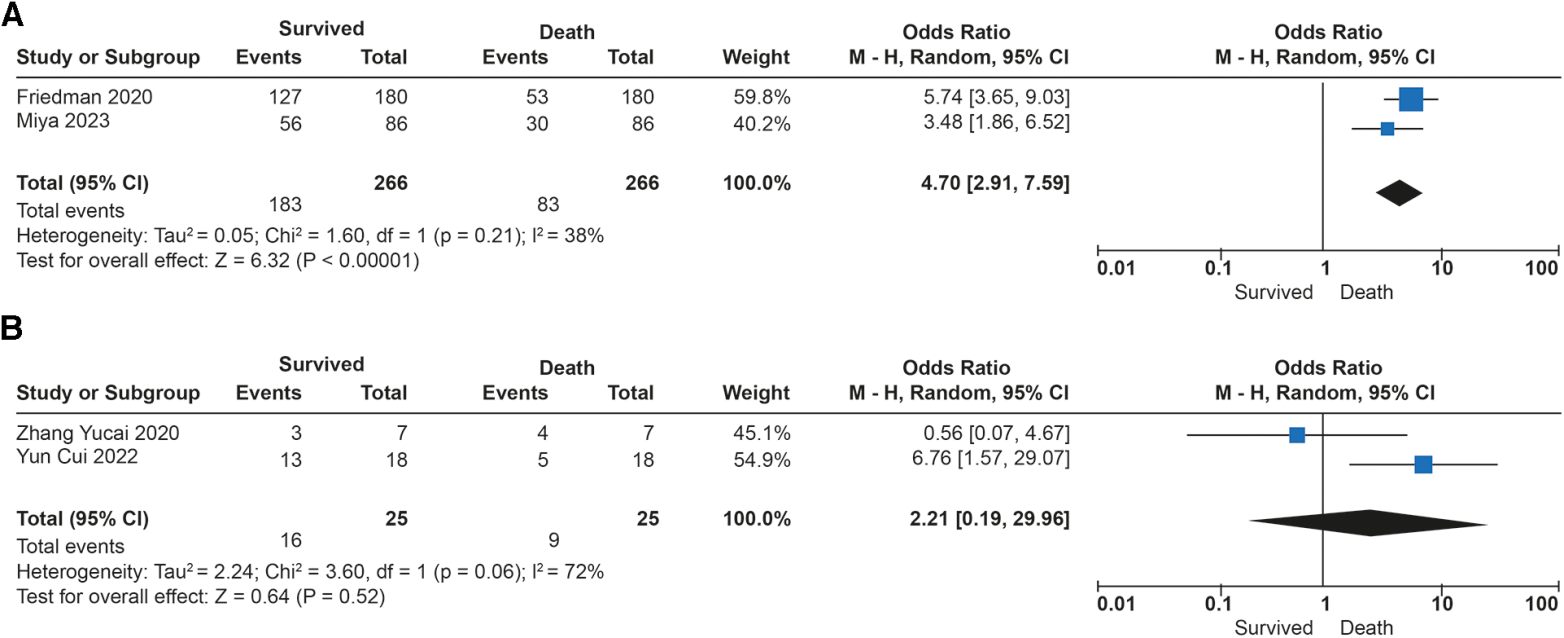
Forest plot de tidal volumen and mortality in patients with ECMO. (**A**) Subgroup analysis with Ultraprotective ventilation (<4 ml/kg). (**B**) Subgroup analysis in patients with protective ventilation (>4 ml/kg).

**Figure 4:**
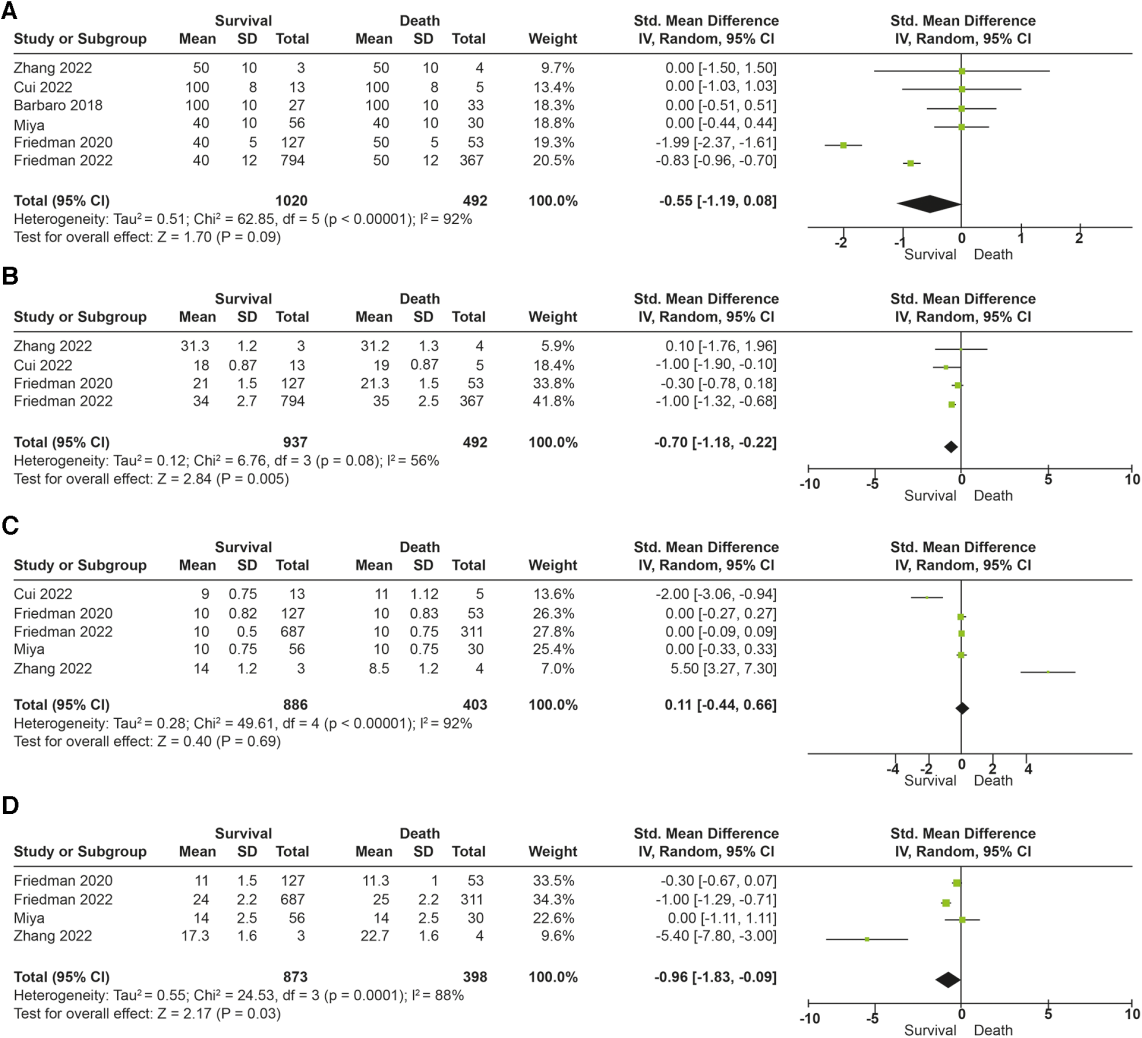
Forest plot parameters of mechanical ventilation and mortality (**A**) fraction of inspired oxygen (%). (**B**) Plateau pressure (cm/H20). (**C**) End expiratory pressure (PEEP). (**D**) Driving pressure (cm/H20).

The association between tidal volume (*V_T_*) and mortality was described in 291 patients. Overall survival in the studies was 69%. No differences in in-hospital mortality were observed in patients receiving low tidal volume ventilation (*V_T_* > 4 ml/kg) (*p* = 0.52). An increased risk of dying was found (OR: 4.70; 95% CI: 2.91–7.59; *p* < 0.01) among children receiving very low tidal volume ventilation (*V_T_* < 4 ml/kg), with low heterogeneity in the studies (*I*^2^ to 38%) (Figures 3A,B). In contrast, patients who survived had lower plateau pressures (mean Dif: −0.70; 95% CI: −0.18, −0.22; *p* < 0.01) compared to those who died. Patients with lower driving pressures (mean Dif: −0.96; 95% CI: −1.83; −0.09; *p* = 0.03) also had lower mortality. No differences were observed in the inspired fraction of oxygen between survivors and those who died (*p* = 0.09). No differences were observed between groups in the PEEP value (*p* = 0.69). The mechanical ventilation parameters included in the analysis are described in Figure 4.

#### Hospital stay and MODS

The survivors had a median hospital stay of 20 days (IQR: 13.2–38.4), and the non survivors had 13.8 days (IQR: 13.2–14.4) (*p* = 0.88). The presence of MODS was described in 152 patients. The group with higher survival had fewer dysfunctional organs compared to patients who died (mean Dif: −1.34, 95% CI: −2.08, −0.60; *p* = 0.01).

Sensitivity analysis was run for the primary outcome. Among the six included studies, Zhang's study (25) differed significantly in terms of the population included because they were exclusively leukemia patients in contrast to the other five studies reporting mechanical ventilation parameters in children with ECMO with sepsis, trauma, and ARDS of other etiologies. However, after excluding Zhang's study, the combined data from the other five studies aligned with each of the outcomes studied. Additionally, high- and low-risk studies were analyzed without finding differences in the overall results. This indicates that our findings were consistent and robust after sensitivity analysis because the results were not affected by the removal of individual studies.

## Discussion

In this systematic review and meta-analysis in children with mechanical ventilation and ECMO, we found that the ventilator parameters associated with higher mortality were very low tidal volume ventilation (<4 ml/kg), high plateau pressure, and high driving pressure. We found no association between mortality and the inspired fraction of oxygen and PEEP provided in MV. Patients who died had more organs involved, with no difference in length of hospital stay.

The main objectives of mechanically ventilated support in patients with ECMO are to limit pulmonary strain, avoid atelectrauma, alveolar overdistension injuries, and resorptive atelectasis (associated with a high inspired oxygen fraction) (26–28). Pulmonary injury associated with mechanical ventilation occurs because of several mechanisms: barotrauma, volutrauma, atelectrauma, ergotrauma, myotrauma, and biotrauma. In large part, this injury is explained by excess strain, or alveolar stress, and shear forces applied during MV (28, 29). Limiting pressures (e.g., plateau airway pressures <30 cm/H20) and *V_T_* (4–8 ml/kg predicted body weight PBW) are often the recommended strategies that have been called pulmonary protective ventilation (14).

Very low tidal volume ventilation was initially suggested by animal researches as a better strategy of protective ventilation. Using a mouse model of acid-induced pulmonary injury, Frank et al. found that reducing the tidal volume from 12 ml/kg to 3–6 ml/kg with the same level of PEEP (10 cm/H20) could reduce mortality (30). It was suggested that decreased pulmonary edema and pulmonary injury might help protect the alveolar epithelium. In adults, research has recently been carried out comparing different tidal volumes in ARDS. Bein et al. compared the strategies of 3 ml/kg PBW vs. 6 ml/kg PBW in 79 adults with ARDS without finding differences in days off mechanical ventilation or mortality between groups (31). Recently, another randomized clinical trial of adults with ARDS and ECMO support evaluated the inflammatory response of very low tidal volume ventilation (2 ml/kg PBW) vs. conventional MV (4–8 ml/kg using the volume control mode). No differences were observed in the concentrations of interleukin-1 beta, interleukin-6, interleukin-8, or surfactant protein D. In fact, the study had to be suspended due to the futility of the results after the inclusion of 39 patients, and higher mortality was observed in the group with very low tidal volume ventilation (45% vs. 17%) (27).

Our findings are consistent with these descriptions. We found that children with *V_T_* less than 4 ml/kg had higher mortality than patients with ECMO, who received low tidal volume ventilation (*V_T_* 4–6 ml/kg). The two main studies that contributed to this finding (19, 26) used pressure-targeted ventilation modes in 90.3% and 77% of the patients, respectively. These patients may have been more seriously ill, with lower respiratory system distensibility. However, these findings could be explained by shear injuries in a severely diseased lung. Additionally, very low tidal volumes can contribute to injury due to cyclic opening and closing of healthy and sick alveolar units, and favor atelectrauma and more VILI (10). This damage occurs especially when the end-expiratory pressure drops below local airway closing pressures (8, 10). In the absence of large, randomized studies in children comparing very low tidal volume ventilation (*V_T_* < 4 ml/kg) with low tidal volume ventilation (*V_T_* 4–6 ml/kg) during ECMO support, a definitive recommendation cannot be made. PALICC-2 mentions that offering lung protective ventilation during ECMO is beneficial, but exact modes and settings remain elusive (15). They recommend maintaining a plateau pressure of less than 25 cm H20 from the start of ECMO support. Our findings suggest that low tidal volume ventilation should be used and other ventilation support parameters considered to decrease driving pressure and mechanical power. In the most severe cases, when DP cannot be decreased, very low tidal volume ventilation could be considered. However, clinical trials are needed to evaluate the safety and efficacy of this strategy.

In this sense, the MV guidelines in pediatric (5, 6, 9, 15) and adult (32) ECMO recommend maintaining adequate levels of PEEP and limiting Pplat but do not give a specific recommendation regarding tidal volume. In our study, high plateau and driving pressure were associated with higher mortality. During MV with ECMO, it is desirable to sustain a Pplat less than 30 cm H20 and preferably look for levels less than 25 cm H20 (12, 15, 32). Low levels of Pplateau have been associated with lower mortality in adult patients with ECMO (28% vs. 46%) (33). In fact, it has also been noted that, in adults, each increase of 1 cm/H20 in the Pplat decreases the in-hospital survival odds by 21% in patients with ARDS and ECMO support (33). Accordingly, with an improved Pplat and optimized PEEP, DP could be reduced, the elevation of which is directly associated with greater mortality. When patients have low DPs at the beginning of ECMO support, it is reasonable to think that they have greater respiratory system distensibility and, therefore, less severe disease, with a greater proportion of functionally uncompromised lung. Chiu Li-Chung et al. recently found that maintaining a DP lower than 21 cm/H20 on the third day of ECMO was associated with lower mortality (56% vs. 33%; *p* < 0.01) compared to patients with higher levels (34). Rambaud et al. found in children that a DP greater than 15 cm/h20 on the first day of ECMO was associated with a higher mortality odds ratio (OR: 2.23, 95% CI: (1.09–4.71), *p* = 0.03) (35). However, they found a higher risk of death with a high PEEP, which we did not find in our study.

Another important finding in our study is the lack of association between Fi02 and PEEP levels and mortality in patients with ECMO. With sufficient oxygenation delivered by the ECMO, it is possible to lower the Fi02 below 40% to avoid further biotrauma. This is not possible for all patients. It is most frequently recommended to maintain Fi02 at 100% in the ECMO at PEEP at the time of its initiation and gradually decrease the Fi02 of the mechanical ventilator below 60%, seeking saturation goals greater than 85% (32, 34). A native lung FiO2 above 60% on D14 has been associated with higher odds of mortality in children on ECMO (OR: 10.36, 95% CI: (1.51–116.15), *p* = 0.03) (35). End-expiratory pressure (PEEP) is a parameter that helps improve functional alveolar units and gas exchange. A tidal volume of 4–6 ml/kg with moderate positive end expiratory pressure (6–10 cm/H20) is the basis of mechanical protective ventilation. Fifty-eight percent of centers with adult ECMO report PEEP use of 6–10 cm/H20, and 22% more than 10 cm/H20 (7, 20). The use of a PEEP adjusted to the condition of each patient, guided by the ARDS net protocol, graphically or with compliance analysis, reduces atelectrauma and prevents alveolar collapse and reopening in each respiratory cycle. The ELSO and PALICC guidelines recommend that patients with ECMO should start with a PEEP of 10 cm/H20, although it is emphasized that it should be adjusted to the condition of each patient (5, 15). The beneficial effects of PEEP may be more related to DP reduction. In a recent meta-analysis of adults, the PEEP level was not related to outcomes or mortality, in line with our findings (36).

We consider that our study has several limitations. First, we found no controlled clinical trials evaluating the safety and efficacy of different ventilatory modes or specific parameters of MV in children with ECMO. We only found observational studies. These studies describe associations that are not necessarily causal. Furthermore, the possibility of residual confounders not being considered in each study having biased the results cannot be excluded. Additionally, we did not find in the studies that the programmed respiratory rate was reported in each patient. In pediatrics, this is an important point because we know that it can vary with age and can be one of the components of ergotrauma, or P-SILI, that affect outcomes. In this regard, patients with *V_T_* < 4 ml/kg had a higher mortality rate. They may have been sicker, with less respiratory distensibility. Thus, DP is directly proportional to *V_T_*. Patients with more severe disease may be the only ones to benefit from very low tidal volume to keep DP as low as possible. In the included studies the majority of patients received VLTV and only twenty-five patients were in the low tidal volume group. This may have limited the findings for this group. No study included considering DP as a variable to adjust tidal volume. Prospective studies are needed to clarify this finding. Some children in the studies were supported with high-frequency mechanical ventilation. We did not find a homogeneous or constant description of parameters in high-frequency oscillatory ventilation (HFOV) to include in the meta-analysis. However, this type of ventilation could help improve alveolar recruitment and some of the outcomes that bias results. In this same sense, we only found studies with veno-venous ECMO. We found no studies with veno-arterial ECMO that specifically described the support parameters in mechanical ventilation, which may limit the extrapolation of our findings. However, in adults with ARDS, the type of cannulation on ECMO is not considered to affect the outcomes of mechanically ventilated patients (31, 32). Finally, we did not find constant descriptions of the duration of mechanical ventilation before the start of ECMO. In experimental studies of adults, this has been shown to be one of the most important factors and determinants of prognosis. Previous mechanical ventilation lasting more than seven days could affect mortality outcomes. We need clinical trials in children that evaluate the impact of these variables on outcomes in children with ECMO.

## Conclusion

This systematic review and meta-analysis found that the variables in mechanical ventilation in children with refractory respiratory failure who receive ECMO support associated with higher mortality are very low tidal volume (<4 ml/kg), high plateau pressures, and high driving pressures. We found no overlap between mortality and other parameters of the mechanical ventilator, such as the inspired fraction of oxygen or the pressure at the end of expiration. Clinical trials in children evaluating the safety and efficacy of the most used MV parameters in patients with ECMO are needed.

## Data Availability

The raw data supporting the conclusions of this article will be made available by the authors, without undue reservation.
